# Biosynthesis of Selenium Nanoparticles (via *Bacillus subtilis* BSN313), and Their Isolation, Characterization, and Bioactivities

**DOI:** 10.3390/molecules26185559

**Published:** 2021-09-13

**Authors:** Asad Ullah, Xian Yin, Fenghuan Wang, Bo Xu, Zulfiqar Ali Mirani, Baocai Xu, Malik Wajid Hussain Chan, Amjad Ali, Muhammad Usman, Nawazish Ali, Muhammad Naveed

**Affiliations:** 1 Beijing Advanced Innovation Center for Food Nutrition and Human Health, Beijing Technology & Business University (BTBU), Beijing 100048, China; asadbahi2016@gmail.com (A.U.); yinxian@btbu.edu.cn (X.Y.); xubaoc@btbu.edu.cn (B.X.); ch.usman1733@gmail.com (M.U.); alibtbu@gmail.com (N.A.); nnourang@yahoo.com (M.N.); 2 School of Light Industry, Beijing Technology & Business University (BTBU), Beijing 100048, China; 3 Food and Marine Resources Research Center, Pakistan Council of Scientific and Industrial Research Laboratories Complex, Karachi 75280, Pakistan; mirani_mrsa@yahoo.com; 4 McIntire School of Commerce, University of Virginia, Charlottesville, VA 22903, USA; 5 Centre of Excellence in Marine Biology, University of Karachi, Karachi 75270, Pakistan; chanwajid@gmail.com (M.W.H.C.); amjadalimb@uok.edu.pk (A.A.)

**Keywords:** selenium, SeNPs, probiotic, *Bacillus subtilis* BSN313, antioxidant, antibacterial

## Abstract

Among the trace elements, selenium (Se) has great demand as a health supplement. Compared to its other forms, selenium nanoparticles have minor toxicity, superior reactivity, and excellent bioavailability. The present study was conducted to produce selenium nanoparticles (SeNPs) via a biosynthetic approach using probiotic *Bacillus subtilis* BSN313 in an economical and easy manner. The BSN313 exhibited a gradual increase in Se reduction and production of SeNPs up to 5–200 µg/mL of its environmental Se. However, the capability was decreased beyond that concentration. The capacity for extracellular SeNP production was evidenced by the emergence of red color, then confirmed by a microscopic approach. Produced SeNPs were purified, freeze-dried, and subsequently characterized systematically using UV–Vis spectroscopy, FTIR, Zetasizer, SEM–EDS, and TEM techniques. SEM–EDS analysis proved the presence of selenium as the foremost constituent of SeNPs. With an average particle size of 530 nm, SeNPs were shown to have a −26.9 (mV) zeta potential and −2.11 µm cm/Vs electrophoretic mobility in water. SeNPs produced during both the 24 and 48 h incubation periods showed good antioxidant activity in terms of DPPH and ABST scavenging action at a concentration of 150 µg/mL with no significant differences (*p* > 0.05). Moreover, 200 µg/mL of SeNPs showed antibacterial reactivity against *Escherichia coli* ATCC 8739, *Staphylococcus aureus* ATCC 9027, and *Pseudomonas aeruginosa* ATCC 25923. In the future, this work will be helpful to produce biogenic SeNPs using probiotic *Bacillus subtilis* BSN313 as biofactories, with the potential for safe use in biomedical and nutritional applications.

## 1. Introduction

Selenium (Se) is an important cofactor for antioxidant enzymes such as glutathione peroxidase and thioredoxin reductase [1,2], and its deficiency can damage the liver, heart, kidneys, skeletal muscle, and testes [3]. However, doses above 400 µg/day can be toxic, and can cause diabetes and prostate cancer [4,5,6]. Se toxicity may arise through mechanisms such as oxidative stress or substitution of selenium for sulfur during protein assembly [7,8]. However, this toxicity depends on the forms of Se. For example, selenium nanoparticles (SeNPs) show better bioavailability and less toxicity compared to inorganic (sodium selenite) forms [9].

Selenium is crucial for the body due to its ability to affect the activity of the selenoenzyme glutathione peroxidase, and to protect cells and tissues from damage, acting as an antioxidant. It is possible that selenium is useful in the prevention of various diseases, including cardiovascular disease, arthritis, muscular dystrophy, and cystic fibrosis [10]. Selenium is widely used as a dietary supplement [11,12,13], particularly because of its relationship with the immune system, and can be used for the treatment of cancer [14]. The interaction of selenium with heavy metals is well known. Selenium compounds are believed to be detoxifying agents, playing an antagonistic role towards mercury, methylmercury [15,16,17,18], cadmium [19], silver [20], lead [21], and many other elements [14].

The specific properties of selenium nanoparticles of various sizes and shapes, compared to Se’s metallic form, have significant application in medicine, including for cancer treatment [22], drug synthesis [23], DNA study [24], magnetic resonance imaging [25], biosensors [26], environmental rehabilitation [27], and pharmaceuticals [28], as well as agricultural [29], electronics, and commercial uses [30].

Selenium nanoparticles (SeNPs) have distinct chemical and physical properties due to their large surface–volume ratio, large surface energy, spatial limitation, and reduced imperfections [31]. The selenium nanoparticles (SeNPs) have numerous applications, particularly in medication, due to therapeutic effects such as low toxicity, better reactivity, low required dosage, and excellent bioavailability compared to other oxidation states of selenium (Se), such as Se^6+^ and Se^4+^ [32,33,34,35]. For the biological activities of Se, SeNPs are regarded as better than the other forms of Se due to their greater biological activity and low toxicity [36].

Nanoparticles can be synthesized by both organic and inorganic means [36]. The biomedical use of inorganic nanoparticles (NPs) has attracted increasing interest in the past few decades. However, in the last couple of years, the interest in nanotoxicology has increased, and more data regarding the cytotoxic properties of inorganic NPs have been reported. Several reviews present an overview of the most important findings on this topic [37,38,39,40,41]. The biogenic nanoparticles are considered to be relatively safe for both human and animal use [42,43]. The microorganisms employ a detoxification mechanism for the reduction of selenites/selenates to nano-selenium, and are referred to as potential biofactories for the synthesis of well-defined selenium nanoparticles [1].

Some comparisons indicate that biogenic techniques are safer and more affordable than other techniques. In non-biogenic techniques, instrumental or chemical methods are used;the instruments required are very expensive [44]. Evaporation and laser ablation techniques are examples of instrumental methods, generally used for the synthesis of nanoparticles [45]. In chemical techniques, additives, solvents, and stabilizers such as borohydride, ethaline, dodecanthiolates, and many other chemicals are applied. These chemicals are not eco-friendly, are hazardous, and are toxic for living beings [44]. Ultimately non-biogenic techniques make the nanoparticles unsafe for biomedical and nutritional uses, while biogenic techniques are safe, inexpensive, eco-friendly, and nontoxic [33,46,47].

The nanoparticles synthesized by microbes have versatile applications and advantages compared to those derived from other, conventional processes [48]. Biogenic SeNPs are more stable, and do not aggregate, owing to the natural coating of the biomolecules [33]. Some microbes—such as yeasts, fungi, and bacteria—are used to produce SeNPs, as they are capable ofsurviving and growing in the selected concentrations of selenium, andcan reduce toxic ions into distinct nanoparticles [49,50]. Bacterial reductions of selenate or selenite occurboth anaerobically and aerobically, by non-enzymatic or enzymatic mechanisms. This biotransformation leads to the formation of cytoplasmic, periplasmic, or extracellular SeNPs [51]. Amongst all of the microbes, bacteria are the best choice for the synthesis of nanoparticles [52], due to their fast growth rate, easy handling, low cost, and high productivity [2]. 

Over the past 10 years, many aerobic and anaerobic bacteria have been reported to have the capability of inorganic selenium (SeO_3_^2−^ and/or SeO_4_^2−^) reduction with the immediate formation of extra/intracellular SeNPs, e.g., *Escherichia coli* ATCC 35,218 [53], recombinant *E. coli* [54], *Ralstoniaeutropha* [24,52], *Enterobacter cloacae* Z0206 [55], *Pseudomonas aeruginosa* ATCC 27,853 [56], *Klebsiella pneumonia* [30,31,57,58], *Pantoeaagglomerans* [14,35], *Zooglearamigera* [2,25], *Rhodopseudomonaspalustris* strain N [59], *Shewanella* sp. HN-41 [60], *Azoarcus* sp. CIB [61], *Burkholderiafungorum* [35,62], *Stenotrophomonasmaltophilia* [36,63], *Staphylococcus carnosus* [37,64], *Lactobacillus casei* [58,65,66], *Lactobacillus acidophilus* LA-5, *Lactobacillushelveticus*LH-B02, *Streptococcus thermophilus*, *Bifidobacterium* BB-12 [65], *Enterococcus faecalis* [67], *Bacillus* sp. MSh-1 [68,69], *Bacillus subtilis* [70], *Bacillus mycoides* SelTE01 [71], *Bacillus licheniformis* JS2 [72], *Bacillus megaterium* [73], *Streptomyces* sp. ES2-5 [74], etc. SeNPs have fascinating potential biological applications, such as antioxidant and antimicrobial uses [75,76,77,78,79,80,81,82]. Among these, the nonpathogenic/probiotic bacteria could be more suitable tools for the production of SeNPs for utilization for medical and nutritional purposes.

In this study, the selenium nanoparticle (SeNP)-producing ability of probiotic *Bacillus subtilis* BSN313 was studied. Produced SeNPs were characterized by UV–Vis spectroscopy, Fourier-transform infrared (FTIR), and morphological studies carried out via scanning electron microscopy (SEM) coupled with energy-dispersive spectroscopy (EDS) and transmission electron microscopy (TEM). The selenium nanoparticles (SeNPs) produced during the 24 and 48 h incubation periods also showed antioxidant activity in DPPH and ABST assays. Moreover, these SeNPs were also tested for antibacterial reactivity against *E. coli*, *S. aureus*, and *P. aeruginosa*. 

## 2. Results

### 2.1. SeNP Synthesis Capability

**Figure 1:**
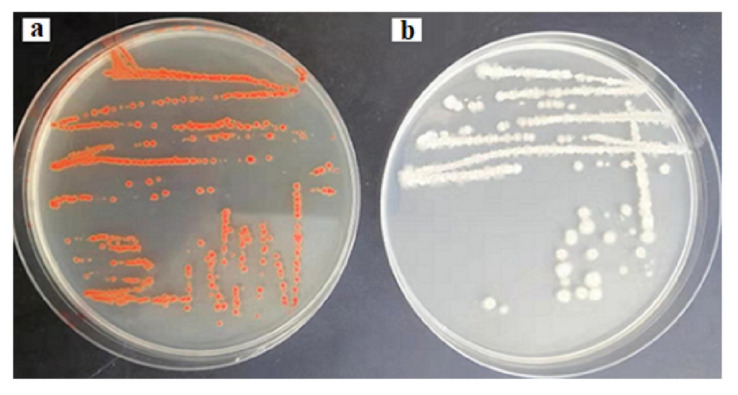
Reddish colonies of BSN313 in the presence of 6 μg/mL Se in Luria Broth (LB) (**a**) and control (**b**).

**Figure 2:**
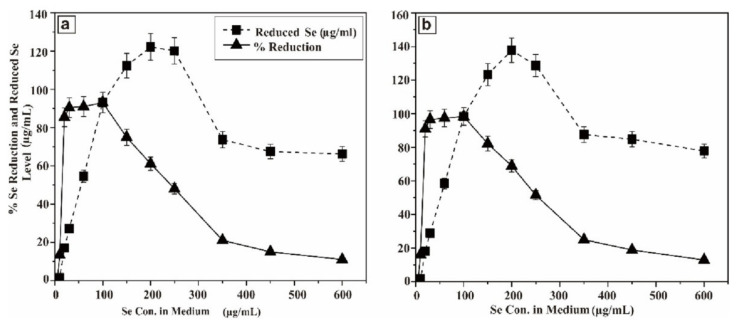
Se reducing capability of BSN313 at 24 h of incubation (**a**) and (**b**) Se reducing capability of BSN313 at 48 h of incubation.

In order to envisage this potential, the probioticwas grown in LB solid agar containing 1–10 µg/mL of selenium as sodium selenite. The appearance of reddish colonies at 6 µg/mL Se (Figure 1a) was the first evidence of selenite reduction, followed by formation of SeNPs. In order to monitor the ability of the strain to produce biogenic SeNPs, it was aerobically (200 rpm) grown for a further 24 and 48 h at 37 °C in liquid LB medium in the presence of a wide range of concentrations of Se (5–600 µg/mL) in the medium. The results are presented in Figure 2. After 5 h, the growing culture was started to turn a red color, which was an indication of the reduction of Se into Se^0^. The significant reddish color was not observed at a low concentration of Se (5 µg/mL), but started to appear gradually at greater concentrations. At a concentration of 100 µg/mL (Se in the medium) 93.1% and 98.33% of Se was reduced into Se^0^ when incubation was continued to 24 and 48 h, respectively. However, the total Se^0^ content in the SeNPs steadily increased, and was found to be higher at 200 µg/mL, though it dropped at higher concentrations. Nevertheless, the strain was able to transform >93.1% of environmental Se (100 µg/mL) in 24 h, despite the SeNP yield being highest at 200 µg/mL.

**Figure 3:**
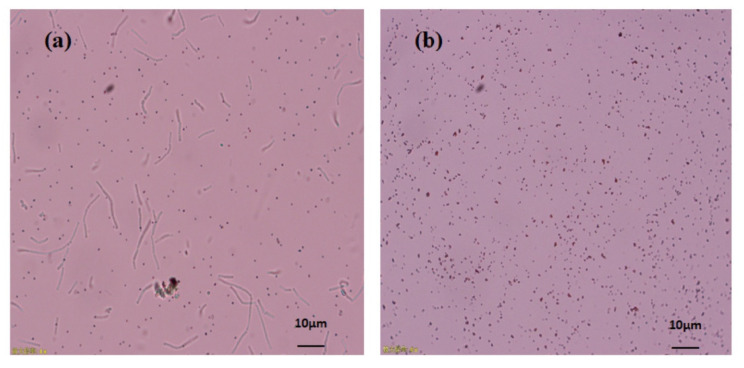
Microscopic image of extracellular SeNPs, before purification (**a**), after purification (**b**).

The proficiency of *Bacillus subtilis* BSN313 in synthesizing extracellular SeNPs was confirmed when it was observed under the microscope at a magnification of ×400 (Figure 3a). At the same magnification, the purification of SeNPs from the cell suspension was also ensured (Figure 3b). These acquired results confirmed that bacterial strain BSN313 effectively reduced Se to elemental Se^0^at the maximum level during the log growth phase. The Se biotransformation capability patterns at both 24 and 48 h were similar, and the SeNP yield was not remarkably high at 48 h (Figure 2). Therefore, SeNPs were produced in bulk for 24 h (200 rpm, 37 °C, and 200 µg/mL Se) for subsidiary characterization. 

### 2.2. Characterization of SeNPs

**Figure 4:**
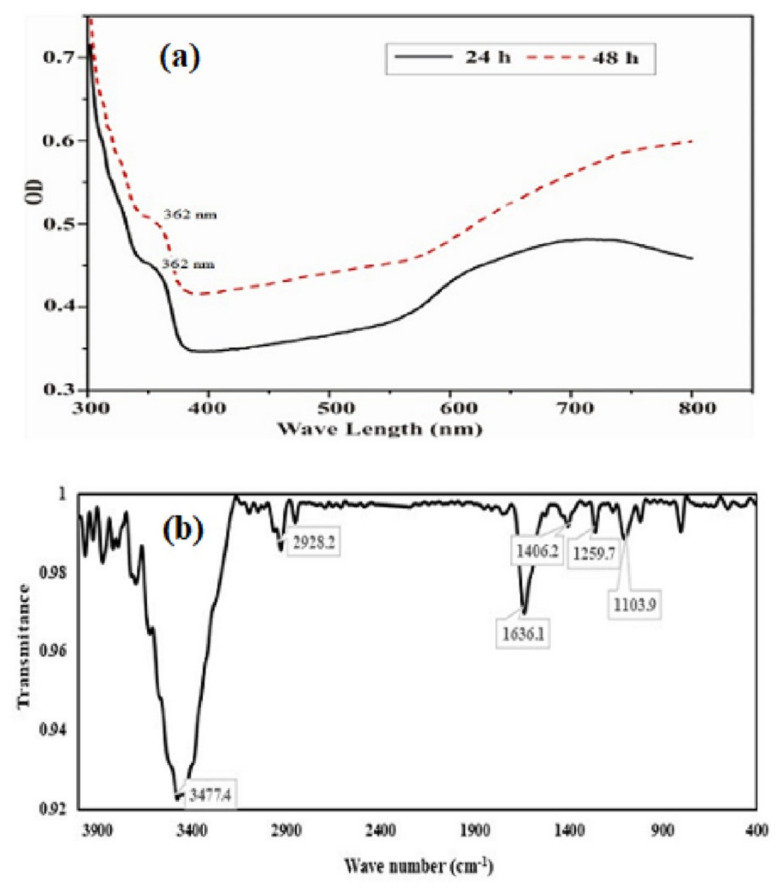
UV/visible (**a**) and FTIR spectra (**b**) of purified SeNPs formed by BSN313 during 24 h.

The UV visible absorption spectrum of purified SeNPs was presented in Figure 4a. The UV-Visible absorption spectra (300–800 nm) of SeNPs produced by BSN313, shown a small transition point at nearby 362 nm and have a maximum absorption peak at 650 nm. The earlier reports for the biosynthesis of SeNPs were also recorded in the literature [83,84]. Fourier transform infrared (FTIR) spectrum of the SeNPs was presented in Figure 4b. Through FTIR, stretching and bending bands of different functional groups (OH, HN, CO, CN), giving the spectral peaks e.g.the peak at 3477 cm^−1^ indicated the OH stretching of the free or intermolecular bonded alcoholic group. The peak at 1406 cm^−1^ may be attributed to the O-H bending of carboxylate. The peak at 1636 cm^−1^ showed the amide N-H bending or any C=O stretch of the ester group while the peak at 1104 cm^−1^evidenced the C-N stretching of the amine as well. The absorption peaks of the NPs may represent to stretching, bending and vibrational frequencies of certain organic functional groups such as NH_2_, COOH, CH_2_ and CO, recorded in the previous literature [85,86].

During biosynthesis of SeNPs carbohydrates and amino groups might be involved, the carbohydrates combine with amino groups bind tightly to the surface of the SeNPs. The use of reducing end chemistry introduces one amino group per carbohydrate (polysaccharide) chain, and provides an excellent way of measuring the exact amount of polysaccharides loaded on the surface of nanoparticle [87].

**Figure 5:**
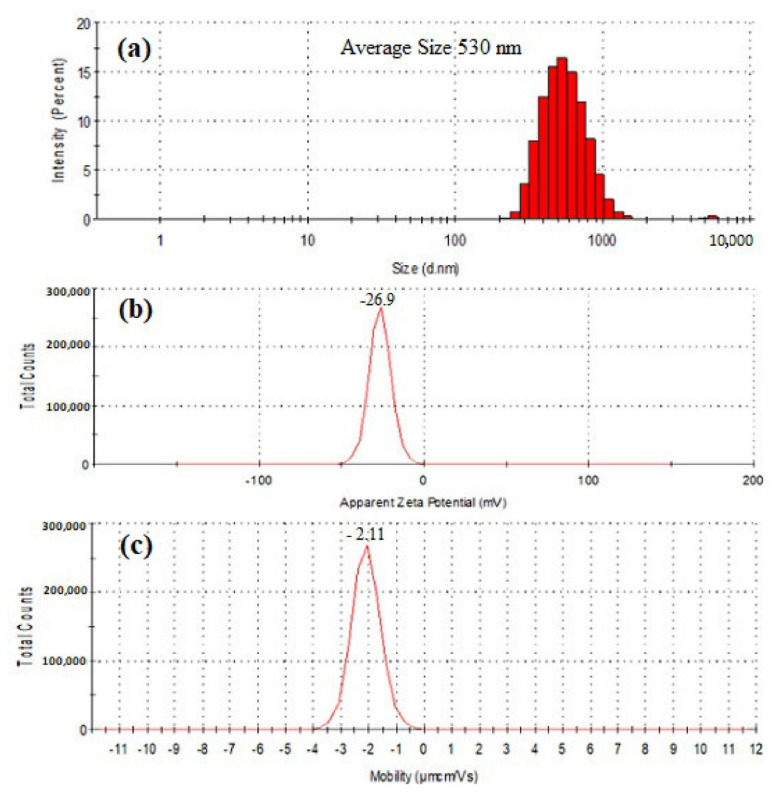
Particle size distribution (**a**), Zeta potential (**b**) and electrophoretic mobility (**c**) of SeNPs produced by BSN313 during 24 h.

The results of particle size, zeta potential (ZP), and electrophoretic mobility (EPM) were presented in Figure 5. The particle size of purified SeNPs was found to be an average of 530 nm when tested by Zetasizer. The zeta potential remains an important indicator of the stability of the colloidal dispersion of nanoparticles. The ZP of freeze dried SeNPs of BSN313 was observed −26.9 (mV) when suspended in deionized water. Zeta potential is the measure of an effective electric charge on the surface of nanoparticles. The nanoparticles with higher magnitude of Zeta potential exhibits increased stability due to greater electrostatic repulsion between nanoparticles. Our result is accordance with the literature [79,88]. 

The negative symbol in the zeta potential means that the net charge of the object is negative. Basic groups have negative charges on their surface [89]. In this study, the SeNPs have amino groups (N-H). The available lone pair of electron on the N-H group makes them electron rich so the net charge remains negative. The attendence of reducing agents (N-H), among the SeNPs, favors them to exist in dispersed form [77]. Same kind of observation was recorded in the previous literature [79,90].

**Figure 6:**
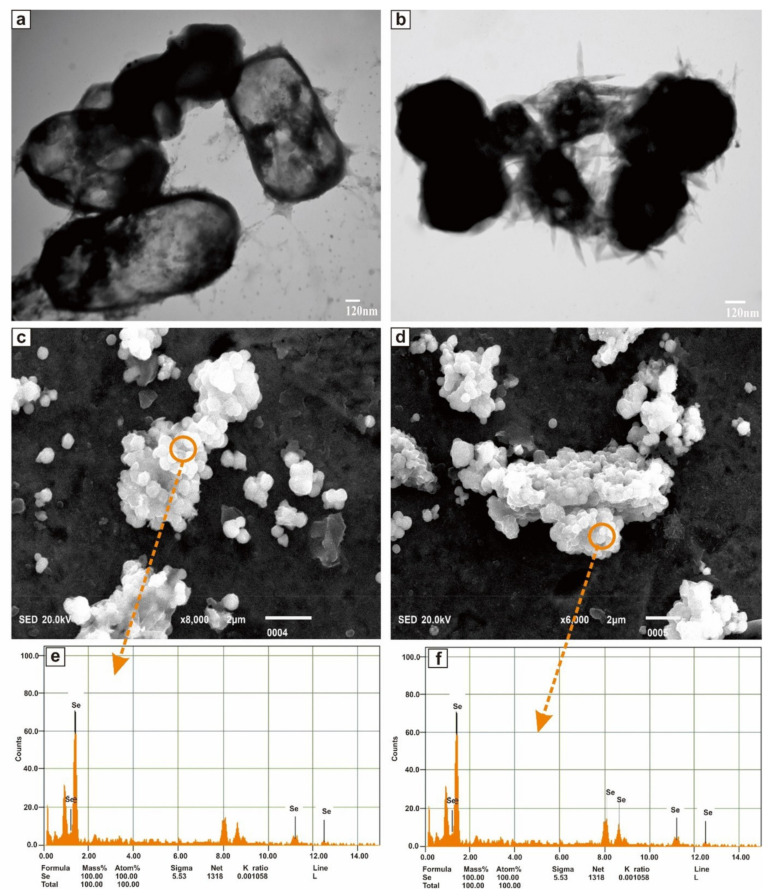
TEM images (**a**,**b**), SEM images (**c**,**d**), and EDS spectra (**e**,**f**) of purified SeNPs produced by BSN313 during 24 h.

The morphology characterization of SeNPs was studied through the TEM and SEM-EDS analysis. Figure 6a,b shown the TEM images of the purified SeNPs at a resolution of 120 nm scale. SeNPs were amorphous and/or spherical in shape and size distribution of manual counting of 50 particles from different TEM images showed that most of the SeNPs were in the range of 280–630 nm. However, the results proved that the nanoparticles have a variable size and were dispersed in an aqueous solution. Figure 6c,d shown the representative SEM micrograph of purified SeNPs, where spherical and/or amorphous shaped Se nanoparticles with some aggregates of different lengths were seen. Elemental analysis of SeNPs was established via the EDS coupled SEM. EDS spectrum was presented in Figure 6e,f which confirmed the presence of Se entirely in the nanoparticles.

### 2.3. Bioactivities of SeNPs

SeNPs produced at 24 and 48 h shown (Table 1) some good antioxidant (DPPH and ABTS scavenging) properties with no significant difference (*p* > 0.05). At the concentration of 150 µg/mL DPPH scavenging ability was found to be 71.25 and 70.40% in 24 and 48 h respectively. At the same concentration, the ABTS free radical scavenging potential was found 63.01 and 60.61% in both 24 and 48h correspondingly.

SeNPs formed at 24 and 48 h also shown a substantial growth inhibition at a concentration of 200 µg/mL against *E. coli* ATCC 8739, *P. aeruginosa* ATCC 25923 and *S. aureus* ATCC 9027 with 11, 12, and 15 mm inhibition zones respectively. Overall, the antimicrobial effect of SeNPs produce at 24 h and 48 h was found not significantly different (*p* > 0.05).

## 3. Discussion

These data suggested that the rate and efficiency of formation of Se^0^ are most likely related to both; the total number of bacterial cells (concentration) and incubation time of the bacterium. However, the incubation period is more important. Delay formation of Se^0^ by improving the growth conditions is the best strategy for maximum microbial Se enrichment. It means, the concentration of Se^0^depends on the incubation period of the microbes. By increasing the incubation time, more enzymatic proteins released by the bacterium, which reduce the ionic selenium (SeO_3_^2−^) to Se^0^ [91,92]. *E. coli* ATCC 35218 was similarly reported to shown 89.2% of Se (1 mM) reduction capability within 72 h of incubation in nutrient broth at 37 ^o^C [53]. In the same way, a decreased in Se concentration (by *K. pneumonia*) was recorded from 200 to 80 ppm in culture media when incubated for 24 h at 37 °C [57]. The microbes transform, ionic Selenium into non-toxic zero valent selenium nanoparticles which can bemore bioavailable selenium source for human and animal nutrition [93]. 

This study is an extension of our previous work, isolation of selenium-resistant bacteria (*Bacillus subtilis* BSN313) from the selenium enriched medium. This probiotic strain has the higher capability to show resistance in selenium enriched medium. BSN313 strain would safely be used as a probiotic tool for the production of SeNPs for nutritional and medicinal purpose [94]. So the present study was designed for developing probiotic nano-selenium containing products/selenium nano-sized particles (SeNPs) via a biosynthetic approach using probiotic *Bacillus Subtilis* BSN313 in an economic and easy way. Characterization of nanoparticles and their therapeutic applications were also analyzed in the present study. The BSN313 exhibited a gradual increase in Se reduction and production of selenium nano-sized particles (SeNPs) from selenium enriched media (600 µg/mL). *Bacillus subtilis* BSN313 reduced the soluble, toxic, colorless selenium ions to the insoluble, non-toxic, red elemental SeNPs.

At the concentration of 100 µg/mL (Se in the medium), more than95% ionic Se was reduced into Se^0^ after 48 h. During the log phase of *Bacillus subtilis* BSN313, the reduction phase (Se^0^) occurred. Same kind of observations were recorded in the same genus, reported in the previous literature [68,70,95]. However, the total reduction occurred, when the concentration of the selenium in the medium was increased to 200 µg/mL. By increasing the selenium concentration in the growth medium, the reduction process began to decrease gradually and stopped at 600 µg/mL.

Morphological changes (nanoparticle size and agglomeration) were observed for SeNPs dispersion by using UV–Vis spectrophotometry. The absorption bands with maxima located between 300-800 nm. The characteristic spectrum was not accurately matched with the previously reported spectra of even SeNPs produced by bacillus species [70,73,96,97]. A number of reports discussed the spectra of SeNPs and confirmed that the distinguish spectral peak of SeNPs was based on chemical arrangement, shape, particle size, and growth conditions [98,99,100,101,102].

In the present study SeNPs synthesis was mediated by *B. subtilis* BSN313. When sodium selenite was added to *B. subtilis* BSN313 culture, the reddish colour indicating the formation of SeNPs [49,103,104]. FTIR spectroscopy is used to confirm the presence of functional groups which were mainly involved in the SeNPs bioreduction process. The FTIR spectrum pattern of SeNPs showed different functional groups at the surface of the SeNPs which may be responsible for the reduction of sodium selenite (inorganic form) to the organic form (SeNPs) [97]. Stretching and bending bands of different groups (OH, NH, CO, CN), confirmed the spectrum of biogenic SeNPs synthesized by *Bacillus subtilis* BSN313. The Broad absorbtion bands, corresponding to the reducing groups (C-O, NH, C-C) present in the bacterial proteins which were responsible for the reduction of sodium selenite into SeNPs. The overall FTIR spectral fingerprint pattern was in agreement with the outline of SeNPs produced by Ramya et al., (2015), Mehta et al., (2021) and Alvi et al., (2021) [105,106,107] via a green synthesis approach. The previous literature indicated, the presence of some biomolecules may have reducing power to synthesize the nanoparticles. Polysaccharides have many functionalities including hydroxyl groups and a hemiacetal reducing end that are capable of reducing precursor salts. The oxidation of polysaccharide hydroxyl groups to carbonyl groups plays an important role in the reduction of selenium salts [108]. The reducing end of polysaccharides can also be used to introduce an amino functionality capable of complexing to and stabilising metallic nanoparticles [109]. Carbohydrates with such amino groups bind tightly to the surface of the SeNPs, giving them a hydrophilic surface. [87,110]. These protein loaded SeNPs, show strong potential against bacterial pathogens also showing antioxidant activity. They play important role in the drugs and widely believed to provide important health benefits [87,111,112,113].

The spherical particles appeared with average size of 530 nm during present study (Figure 5a). It was an agreement from the previous literature [49,65]. These particles are charged species, the charge is measured by zeta potential. Zeta potential value other than −30 mV to +30 mV (depending on the charge) is generally considered to have sufficient repulsive force for the nanoparticles, to remain in the colloidal system. On the other hand, a small zeta potential value can result in particle aggregation and flocculation due to the van der Waals attractive forces act upon them [114,115]. During this research, the recorded zeta potential (−26.9 mV) indicates that these nanoparticles aggregated quickly in the solutions leading to a stable dispersion and can be easily obtained. Same kind of approach was recorded by Fritea et al., (2017) [116]. Moreover, the EPM of SeNPs in water was found −2.11 µm.cm/Vs. EPM measurement is a powerful technique to estimate the surfaced electrical properties of a charged spherical colloidal particle in a solution [117,118]. The high negative charge on the surface of the nanoparticles could also be an indication of the greater stability of the biogenic nanoparticles [50].

The stability of nanoparticles is not only important during any process or treatment but also for their respective function [119]. As the SeNPs purification procedure included alternative sonication (300 W) with serial treatment followed by washing with various buffers and salt solutions [73]. This technique was used in the present study in order to maintain stability and durability of obtained product (SeNPs). 

Spherical and/or amorphous shaped SeNPs, with some aggregates of different lengths were recorded. Same kind of SeNPs were reported in the previous literature for most of the bacteria [120,121,122]. The sizes and shapes of biogenic metallic nanoparticles can be controlled by exchanging the bio-reduction conditions, including type of culture and organism, nature of the medium and incubation time etc. [120]. The size and shape of the SeNPs were confirmed by transmission electron microscope (TEM) Spherical/amorphous SeNps, deposited on the surface of *Bacillus Subtilis* BSN313 (Figure 6a,b). The process of formation of SeNPs (spherical/amorphous) was in agreement with Srivastava et al., (2013) [2]. Scanning electron microscopy with energy dispersive X-ray spectroscopy (SEM-EDS) is a widely accepted technique for the analysis of these biogenic particles, with the presence of selenium in them and display spectroscopic maps by showing the elemental distribution of SeNPs obtained by TEM imaging. The recorded image from SEM-EDS (Figure 6c,d), showing the elemental distribution of Se in the SeNPs.

Selenium nanoparticles (SeNPs) are attracting much attention for their excellent biological activities and low toxicity. Many studies have revealed that the SeNPs exhibited novel antioxidant activities in vitro and in vivo by the activation of selenoenzymes [34,123,124,125,126,127]. In the present study, SeNPs produced at 24 and 48 h shown (Table 1) some good antioxidant (DPPH and ABTS scavenging) activities. Exopolysaccharide-capped SeNPs synthesized by *Bacillus paralicheniformis* SR14 were also found with better antioxidant properties on scavenging DPPH and ABTS free radicals at the concentration ≥1 mM [128]. Whereas, Forootanfar et al., (2014) [68] reported the moderate DPPH and ABTS free radicals scavenging effect of SeNPs produced by *Bacillus* sp. MSh-1 at the concentration of ≥120 µg/mL. In the same way, Greeshma and Mahesh, (2019) [129] reported SeNPs, emerged via *Bacillus* species as good antioxidant substitutions by DPPH assay at the IC_50_ value of 11.6 µg/mL.

SeNPs formed at 24 and 48 h also shown a substantial growth inhibition at a concentration of 200 µg/mL against *E. coli*, *P. aeruginosa* and S. *aureus*. Srivastava and Mukhopadhyay, (2015) [52] reported, 99% inhibition of SeNPs at 250 µg/mL against, *E. coli*, *P. aeruginosa*, *S. aureus* and *S. pyogenes*. Similarly, Geoffrion et al., (2020) [130] also reported SeNPs as good antimicrobial agents against MDR *E. coli*, *P. aeruginosa*, S. *epidermidis*, and MRSA. Likewise, SeNPs produce via *B. amyloliquefaciens* SRB04 had shown a remarkable antibacterial activity on S. *aureus* compared with broad-spectrum antibiotic chloramphenicol [97]. It is already discussed in detail that the surface of the SeNPs is loaded by carbohydrates plus protein complex structure, showing strong potential against bacterial pathogens also having antioxidant activity.

The antioxidant and antibacterial activities of SeNPs synthesized by other species of *Bacillus* strain were also recorded. The SeNPs synthesized by *B. amyloliquefaciens*, have no bactericidal activity against *E. coli* PTCC 1329 while giving significant antibacterial activity against *S. aureus* PTCC 1112 (ZOI: 18.6 mm) [97]. The SeNPs synthesized by *B. laterosporus*, have higher zone of inhibition (ZOI) against *E. coli* (22 mm) and *S. aureus* (37 mm) [131]. The nanoparticles made by *Bacillus cereus* and *Bacillus* species Msh-1 have antioxidant activity [50,132]. 

## 4. Material and Methods

### 4.1. Equipment Used

Shaking incubator (Model No. IFORS AG CH-4103) made in Bottmingen Switzerland, High-Pressure Steam Autoclave (Model No. HVA-100) made by Hirayama, Japan. pH Meter (Model No. FE20) equipped with InLab Pure Pro-ISM probe made by Mettler Instruments Shanghai Co. Ltd. Freeze dryer (Model No. ALPHA 1-4 LSC) made by Martin Christ. Multimode Microplate Reader (Model No. Infinite M200 PRO) made by Tecan in Switzerland, Microscope (Model No. CX31 Japan Olympus Corporation), FTIR (FTS-65, Bio Rad, USA) Zetasizer Nano Series (Malvern, UK). Scan Electron Microscope coupled with EDS (JEOL JSM-IT 100, Japan); Transmission Electron Microscopy (JEOL JEM-1010, JEOL, Peabody, MA, USA).

### 4.2. Supplies and Chemicals

Analytical grade Na_2_SeO_3_ (≥99.0%), HCl (≥37%), Glucose anhydrous (≥99.0%), Sucrose anhydrous (≥99.0%), NaCl (≥99.0%), KCl, MgSO_4_×7H_2_O (≥98.0%), TrisHCl (≥99.0%), NaOH (≥98.0%), Na_2_S(≥99.0%), Na_2_HPO_4_×12H_2_O (≥99.0%) and KH_2_PO_4_ (≥99.5%), were purchased from Sinopharm Chemical Reagent Co. Ltd, China. Biological grade peptone was acquired from Beijing Aoboxing Biotechnology Co. Ltd. Biological grade agar was produced by the Beijing Kangbeisi Technology Co. Ltd. Yeast Extract LP0021 was from Oxoid, made in France. Potassium persulfate (≥99.0%) and 2,2-Diphenyle-1-picrylhydrazyl (DPPH, ≥95.0%) were purchased from Sigma-Aldrich.

### 4.3. Preparation of Common Solutions and Medium

A stock solution of Se (50 mg/mL) was prepared by exactly weighing 5.475 g of sodium selenite (Na_2_SeO_3_), dissolved and diluted up to 50 mL with deionized water, and the solution was sterilized by passing it through a 0.22 µm syringe filter. 

LB (Luria Broth) was prepared by adding 0.5 g yeast extract, 1 g peptone, and 1 g NaCl, to 100 mL distilled water and adjusted the pH to 7.0, consuming 1 M HCl. LB solid medium was prepared by adding 1.5g agar to the LB medium and autoclaved at 121 °C for 15 min. An appropriate volume of hot medium was poured into sterilized petri plates and then allowed to solidify. The required concentration of Se was achieved by adding filtered sterilized stock solution in hot LB medium. 

### 4.4. SeNPs Synthesizing Capability

#### 4.4.1. Strain Activation and Preparation of Inoculum

*Bacillus Subtilis* BSN313previously isolated from traditional Chinese “Jiuqu” [94] was used in this study.Strain (frozen at −80 °C) was thawed on ice and streaked on LB medium plates, by means of an inoculation loop and incubated at 37 °C for 12 h. A single colony was inoculated into a pre-sterilized 10 mL tube containing 4 mL of LB broth and grown at 37 °C for 12 h at 220 rpm shaking speed.

#### 4.4.2. Selenium Reducing Capability

Initially, the inoculum was streaked on LB medium with (1–10 µg/mL) and without selenium (control) in order to check the Se reducing capability. The appearance of reddish colonies in the presence of Se was the first evidence for Se reducing ability.

Added 1 mL of inoculum in a 250 mL flask containing 100 mL of sterilized LB medium. Supplemented a required volume of sodium selenite (50 mg/mL) solution to contribute 5,10, 20, 30, 60, 100, 150, 200, 250, 350, 450 and 600 μg/mL of Se in the medium. Grown in shaking incubator at 37 °C for 24 and 48 h at 200 rpm. The total reduce Se (S^0^) was determined in each flask and calculated the percent Se reduction capability of BSN313.

The production of extracellular selenium nano-spheres (SeNPs as S^0^) were preliminarily confirmed by color (reddish) change followed by observing the culture suspension (Figure 3a) under the microscope (Model No. CX31 Japan Olympus Corporation).

#### 4.4.3. Determination of Reduced Selenium

Exactly, 20 mL of bacterial red-coloured culture was taken from the flasks comprising different Se concentrations (5 to 600 μg/mL). Centrifuged at 8000 rpm for 10 min and in order to remove non-metabolized selenite, the pellets were washed twice with 20 mL of 1M NaCl. The red colloidal Se in the pellet was dissolved in 2 mL of 1M Na_2_S and then centrifuged to eliminate bacterial cells debris. A calibration curve (R^2^ = 0.995) was made using the method adopted by Mishra et al., (2011) [73] with some modifications. 125-950 μg of seleniumas sodium selenite was chemically reduced using 40 μL of 1 M HN_2_OH·HCl in a total of 400 μL reaction system volume. The contents were allowed to stand for 1 h 37 °C temperature and then dried under the stream of N_2_. Add 2 mL of 1 MNa_2_S solution to each tube, mixed and the intensity of the red-brown was read at 500 nm.

#### 4.4.4. Final Production of SeNPs

SeNPs were prepared (in bulk for characterization/application) in 1 L shaking flask containing 250 mL LB medium along with 200 μg/mL of selenium. A 1 mL of BSN313 inoculum was added and allowed to grow in a shaking incubator at 37 °C and 200 rpm for 24 h. 

SeNPs were purified by the procedure adopted by Mishra et al., (2011) [73] with some amendments. The red colour bacterial culture was centrifuged at 8000 rpm for 5 min and collected the pellet. Added 30 mL of 0.5M NaCl to proximately 1 g pellet and ultra-sonicate at 300 W for 10min. Centrifuge for 5 min at 8000 rpm. The pellets were re-suspend and centrifuge successively in 0.5M NaCl, 0.5 M sucrose, and finally a complete salts solution containing 17.5 g NaCl, 0.74 g KCl, 12.3 g MgSO_4_×7H_2_O and 0.15 g of TrisHCl per liter, adjusted the pH to 7.5. The cells were lysed in 30 mL of 0.057% lysozyme solution in complete salts. The lysed cells were washed away from the nanoparticles by sequential re-suspension and ultra-sonication and centrifugation (8000 rpm) in 30 mL of complete salts solution, 0.25M NaOH, 0.1M NaOH, 10 mM Na_2_HPO_4_ (pH 7.3), distilled and deionized water. Lastly, the SeNPs were re-suspended in deionized water. To ensure the purification of SeNPs, final solution was observed in microscope (Figure 3b) at a magnification of 400×. For further characterization, the purified SeNPs were freeze dry at 0.12 mbr and −40 °C for 24 h. 

### 4.5. Characterization of SeNPs

#### 4.5.1. UV/visible and FTIR Spectral Analysis

Suspension of purified SeNPs was made in deionized water and then the UV visible spectra was recorded range between 200–800nm using a multimode microplate reader (Infinite M200 PRO). While FTIR spectra (4000~400 cm^−1^)was noted through FTS-65, Bio Rad using freeze-dried powder of purified SeNPs.

#### 4.5.2. Particle Size, Zeta Potential and Electrophilic Mobility Measurements

Zeta Potential (ZP), size distribution, and electrophilic mobility (EM) were conducted through Zetasizer Nano Series (Malvern). SeNPs were dispersed in deionized water and sonicated for 8 min then around 0.5 mL of the suspension was transferred to the cuvette of dip cell kit for particle size distribution ZP and EM.

#### 4.5.3. Transmission Electron Microscopy

JEOL JEM-1010 Transmission Electron Microscope (TEM) was used to understand the morphological appearance. Purified SeNPs were suspended in deionized water and deposited a drop of suspension on a carbon-coated copper grid and dried at room temperature. TEM was operated to visualized the SeNPs at the accelerating voltage of 100 kV at 0.4 nm point to point resolution. 

#### 4.5.4. Scan Electron Microscopy EDS

Scanning electron microscopy (SEM) coupled EDS was used to morphological and elemental composition of SeNPs. Nanoparticles (Se) were sterilized by ultraviolet light in laminar air flow. The sterilized nanoparticles were carefully mounted on SEM stubs by using adhesive tape and uniformly coated with carbon (JEOL-EC-32010CC) and placed in a sample chamber of SEM-EDS (JEOL JSM-IT 100, Japan) and scanning was performed under different magnifications—ranging from ×6000 to ×8000 —and a voltage of 20 kV.

### 4.6. Bioactivities of SeNPs

#### 4.6.1. DPPH Scavenging Assay

For the DPPH scavenging assay, the method of Lee et al., (2015) [82] was followed with some amendments. Briefly,150 μL of DPPH solution (0.08 mg/mL ethanol) and 50 μL sample (150 µg/mL) were mixedin a well of96-well plate (in triplicate). 50 μL distilled water was used instead of the sample as a control group, and anhydrous ethanol was used instead of DPPH solvent as a blank group. The plate was kept in dark for 90 min and then absorbance was measured at 514 nm.

(1) $$\mathrm{DPPH}\;\mathrm{free}\;\mathrm{radical}\;\mathrm{scavenging}\;\mathrm{rate}\;\left(\%\right)=\frac{A\;\mathrm{control}\;\mathrm{group}\;-\;A\;\mathrm{sample}\;\mathrm{group}}{A\;\mathrm{control}\;\mathrm{group}\;-\;A\;\mathrm{blank}\;\mathrm{group}}\times 100$$

#### 4.6.2. ABTS Scavenging Assay

ABTS scavenging assay was performed using the method adopted by Lee et al., (2015) [133] with some modifications. Briefly, 7 micro moles ABTS was made in a solution of potassium persulfate (140 mM), protected from light for 10 min, diluted with PBS (pH 7.4, 0.1M) to its absorbance 1.0 ± 0.02 at 734 nm. A 150 μL ABTS was placed in per well of 96-well plate (in triplicate) and added 50 μL sample (150 µg/mL). 50 μL distilled water was used instead of the sample as a control group and 50 μL of ABTS solvent (PBS, pH 7.4, 0.1M) was used as a blank group. The plate was kept in dark for 40 min and absorbance was measured at 734 nm.

(2) $$\mathrm{ABTS}\;\mathrm{free}\;\mathrm{radical}\;\mathrm{scavenging}\;\mathrm{rate}\;\left(\%\right)=\frac{A\;\mathrm{control}\;\mathrm{group}\;-\;A\;\mathrm{sample}\;\mathrm{group}}{A\;\mathrm{control}\;\mathrm{group}\;-\;A\;\mathrm{blank}\;\mathrm{group}}\times 100$$

#### 4.6.3. Antimicrobial Activity

The antibacterial activities of the SeNPs were examined against *E. coli* ATCC 8739, *S. aureus* ATCC 25923, and *P. aeruginosa* ATCC 9027 in tryptone soya agar (TSA). The overnight cultures of the subject isolate were inoculated on TSA Plate. Various concentrations (100, 150 and 200 µg/mL) of SeNPs were applied. The inhibition zone around SeNPs spots were measured after 24 h of incubation at 35 °C.

## 5. Conclusions

A growing need for sustainability initiatives in the field of nanotechnology has brought about the development of biogenic procedures for the synthesis of SeNPs—a development which is rapidly replacing traditional chemical syntheses. This transition has many advantages, including the decreased use of chemicals that are toxic to our health and the environment, and the creation of a collection of nanocomposites with many novel applications in nanobiotechnology. The characterization and bioactivities of SeNPs confirmed adequate composition and viability of the produced SeNPs against bacterial pathogens, as well as good antioxidant activity. The results of this study could have a great impact due to the simple culture requirements for BSN313, with an accordingly low production cost of biologically important SeNPs. Therefore, with these potential biological impacts, the tested SeNPs have a tremendous potential for application in the pharmaceutical, biomedical, and food industries, and especially as antimicrobial and antioxidant agents. Further studies are necessary in order to demonstrate possible medical and biological applications, such as in food supplements and pharmaceuticals.

## Figures and Tables

**Table 1:** Antioxidant and antibacterial effects of SeNPs produced by BSN313.

	24 h	48 h
DPPH (%)	71.25 ± 2.81 ^a^	70.40 ± 2.67 ^a^
ABTS (%)	63.01 ± 2.74 ^a^	60.61 ± 3.03 ^a^
*E. Coli* ATCC 8739 (mm)	11.3 ± 0.58 ^a^	11.3 ± 0.58 ^a^
*P. aeruginosa* ATCC 25923 (mm)	12 ±1.0 ^a^	12 ± 1.0 ^a^
*S. aureus* ATCC 9027 (mm)	15 ± 1.0 ^a^	15 ± 0.58 ^a^

^a^ Data with the same superscript letters indicate no significant differences (*p* > 0.05), *n* = 3.

## Data Availability

The data is available on request.
